# The Prognostic Value of Platelet-to-Lymphocyte Ratio in Urological Cancers: A Meta-Analysis

**DOI:** 10.1038/s41598-017-15673-2

**Published:** 2017-11-13

**Authors:** Dong-Yang Li, Xuan-Yu Hao, Tian-Ming Ma, Hui-Xu Dai, Yong-Sheng Song

**Affiliations:** 1 Department of Urology, Shengjing Hospital of China Medical University, Shenyang, Liaoning 110004 P.R. China; 2 Department of Rheumatology and Immunology, Shengjing Hospital of China Medical University, Shenyang, Liaoning 110022 P.R. China; 3 Department of Clinical Epidemiology and Evidence-based Medicine, The First Hospital of China Medical University, Shenyang, Liaoning 110001 P.R. China

## Abstract

The relationship of platelet-to-lymphocyte ratio (PLR) and survival in urological cancers remained inconsistent in previous studies. Therefore, we performed a meta-analysis to assess the prognostic significance of PLR in patients with urological cancers. A literature search was performed in the PubMed, Embase, and Web of Science up to July, 2017 and study quality was obtained using the Newcastle-Ottawa Scale. To estimate the association of PLR and overall survival (OS) and other survival outcomes in urological cancers, we used pooled hazard ratios (HRs). Subgroup analyses were conducted on different ethnics, sample sizes and cut-off values. 20 high quality studies involving 7562 patients with urological cancers were included in this meta-analysis. High pretreatment PLR was significantly associated with poor OS in patients with urological cancers (pooled HR = 1.58). Elevated PLR was also correlated with other survival outcomes. However, we found that PLR was significantly relevant to the OS of patients with different types of urological cancers except bladder cancer (BCa, HR = 1.16, 95%CI: 0.96–1.41). In conclusion, elevated PLR was negatively related to the OS of patients with urological cancers, except in BCa. However, more large scale prospective studies with high quality are required in the future.

## Introduction

Urological cancer contains major malignancies with high morbidity and mortality worldwide^1^. Prostate cancer (PCa) is the most prevalent cancer among western men, accounting for 19% new cancer cases and third leading cause of cancer-related death among American men in 2017^2^. Bladder cancer (BCa) represents the ninth most common tumor with approximately 2.7 million people suffering from it on a global scale^3,4^. While Renal cell carcinoma (RCC) takes up for a proportion of 2–3% in overall adult cancers^3^. Upper urinary tract urothelial carcinoma (UTUC) is relatively rare but aggressive. Despite great progress of treatment, the prognosis and clinical outcome of urological cancers remains unsatisfactory because of local recurrence or distal metastasis. The survival rate and survival time may be increased by risk stratification and optimal treatment at early stage. Hence, it is crucial to seek useful personalized biomarkers to estimate patient prognosis.

Accumulating evidence demonstrates that inflammation plays a critical role in tumor development and progression^5,6^. In patients with cancer, systemic inflammation is likely to affect the tumor micro-environment and promote tumor growth, which means poor outcome^7^. It is well acknowledged that systemic inflammation response can be characterized by the changes of peripheral blood cell amounts. Numerous studies have revealed that blood-based biomarkers show great potential in urological cancer prognosis, such as neutrophil-to-lymphocyte ratio (NLR), C-reactive protein (CRP), lymphocyte-to-monocyte ratio (LMR) and platelet-to-lymphocyte ratio (PLR)^8–10^. Platelet and lymphocyte counts are easily acquired during routinely blood tests in clinical laboratories worldwide. Thus, PLR is a cheap and objective parameter to potentially help doctors assessing patient prognosis. A couple of meta-analyses have testified the prognostic impact of PLR in malignancies like breast cancer and lung cancer^11,12^.

During the past few years, several studies explored the prognostic significance of PLR in patients with urological cancers. However, some studies have drawn contradictory conclusions. To our knowledge, no published meta-analysis have investigated PLR and urological cancer prognosis. The aim of the current study was to quantitatively and comprehensively summarize the available evidence on the prognostic value of elevated PLR and different survival outcomes (overall survival, OS; cancer specific survival, CSS; progression free survival, PFS and disease free survival, DFS) in patients with urological cancers.

## Results

### Study search and characteristics

**Figure 1:**
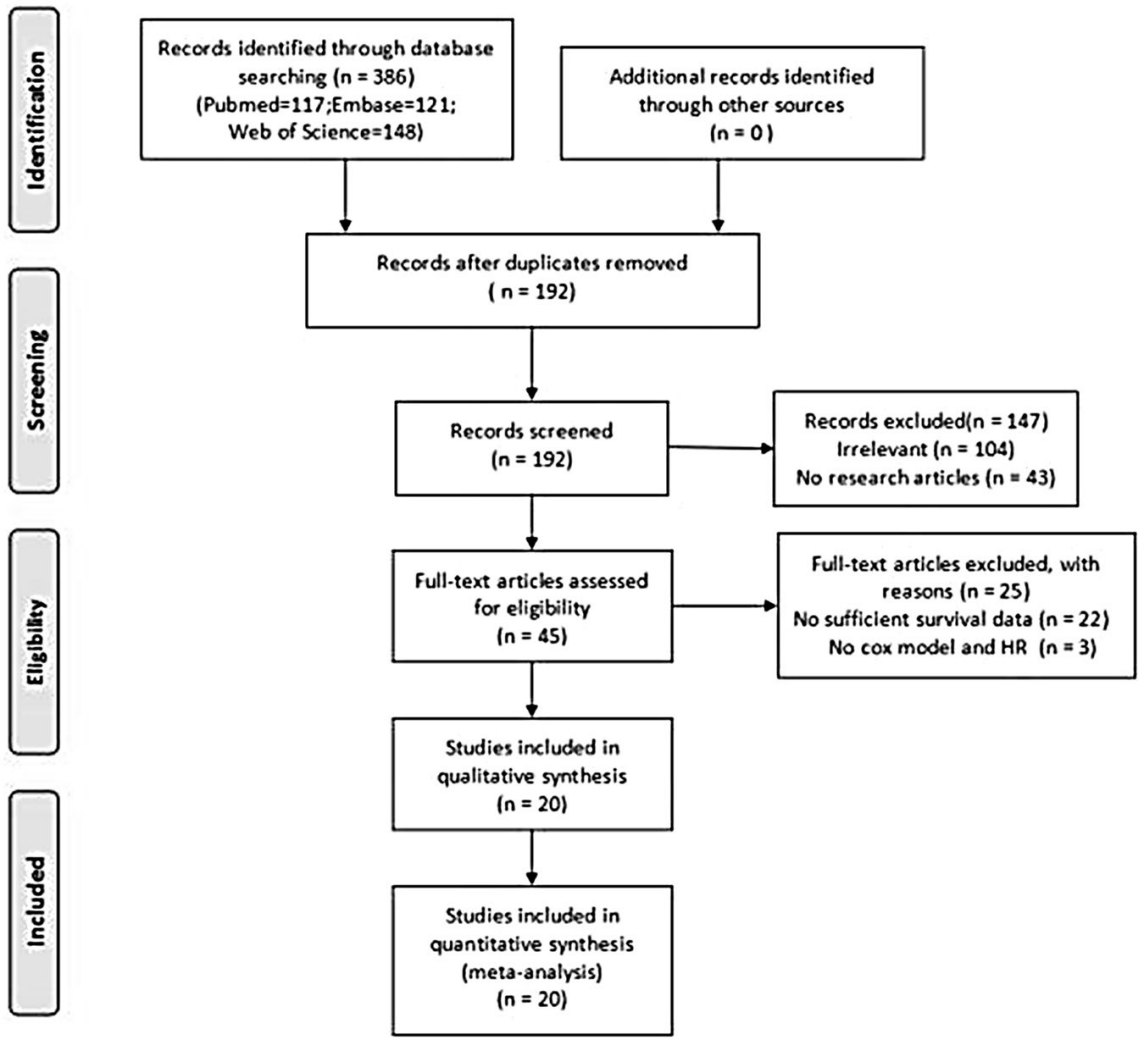
Flow chart of literature search and study selection.

We performed literature search under the guideline of Preferred Reporting Items for Systemic Reviews and Meta-Analyses (PRISMA) statement^13^. The process of literature selection was shown in a flow diagram (Fig. 1). A total of 386 studies were initially identified with the keywords used to search the databases. By screening the titles and abstracts, 45 potential studies were retrieved. 25 studies were then excluded after further fully reviewed because they were insufficient of data (22 studies) or didn’t use cox model and hazard ratio (HR, 3 studies^14–16^). Finally, 20 cohort studies^17–36^ met the inclusion criteria for our meta-analysis.

The baseline information of the studies was shown in Table 1. The articles were published from 2013 to 2017, including 7562 patients, highlighting the recent interest of investigating this topic. Of them, 12 cohorts^17,19,21–23,26,27,29,31,33,34,36^ were from Asian countries and 8 cohorts^18,20,24,25,28,30,32,35^ were non-Asian. Overall, 17 studies investigated the prognostic ability of PLR and OS, while 4 studies discussed PLR and CSS^19,20,28,29^, 4 articles studied PLR and PFS^19,20,33,36^, and other 4 studies reported PLR and DFS^29–32^ in patients with urological cancers.

**Table 1:** Baseline characteristics of included studies.

Study ID	Country	Duration	Cancer type	Sample size	Median age (years)	Sex (male)	Median follow up (months)	Cut-off value	HR of OS	95% CI	Multivariate analysis	Study quality (NOS score)
Li *et al*.^17^	China	Dec. 2009–Jun.2012	PCa	103	66	100%	36	150	2.41	1.61–4.73	yes	8
Lolli *et al*.^18^	Italy	Apr. 2011–May 2015	PCa	230	74	100%	29	210	1.41	0.97–2.03	yes	7
Wang *et al*.^19^	China	Jan. 2010–Dec.2014	PCa	290	75	100%	37	117.58	1.65	1.013–2.687	yes	7
Langsenlehner *et al*.^20^	Austria	1999–2007	PCa	374	68	100%	87	190	1.87	1.02–3.42	yes	7
Sun *et al*.^31^	China	2011–2016	PCa	171	68.5	100%	40	134	2.1	0.8–4.9	no	6
Martinez *et al*.^32^	Spain	Jan.2012–Nov.2015	PCa	101	73	100%	25	150	2.21	1.18–4.12	no	6
Hu *et al*.^21^	China	Jan. 2006–Jun.2010	RCC	484	56	57.40%	36	185	1.64	0.88–3.05	yes	7
Gu *et al*.^22^	China	Jan. 2004–May.2015	RCC	103	56	68.90%	19.9	132	1.172	0.618–2.220	yes	7
Park *et al*.^23^	Korea	2007–2013	RCC	63	63.1	82.50%	17.5	150	16.1	4.4–58.4	yes	6
Fox *et al*.^24^	Australia	Dec. 2002–Feb.2005	RCC	362	62	74.03%	12	195	1.88	1.48–2.37	no	7
Gunduz *et al*.^25^	Turkey	May 2009–Sep.2013	RCC	100	58	71%	32.7	210	1.445	0.949–2.200	yes	7
Peng *et al*.^33^	China	2001–2010	RCC	1360	55	70%	60	164.33	1.19	0.83–1.71	no	6
Zhang *et al*.^26^	China	Jan. 2009-Dec.2009	BCa	124	65	80.65%	36	125	1.161	0.605–2.226	no	7
Kang *et al*.^34^	Korea	1990–2013	BCa	1,551	65	83.90%	52	124	0.99	0.76–1.31	yes	7
Schulz *et al*.^35^	Germany	2004–2015	BCa	665	70	77%	27	28	1.4	1.0–1.8	yes	7
Huang *et al*.^27^	China	Jan. 2002–Jun.2013	UTUC	481	65.8	64.70%	40	241.2	1.61	0.94–2.76	yes	7
Dalpiaz *et al*.^28^	USA	Sep. 1990–Jul.2012	UTUC	180	70	60.60%	30	150	1.782	1.041–3.050	yes	7
Kim *et al*.^29^	Korea	1999–2010	UTUC	277	63.7	78.70%	57.2	150	NR	NR	yes	7
Altan *et al*.^36^	Turkey	since 1990	UTUC	113	63.7	86%	34	150	NR	NR	yes	7
Lucca *et al*.^30^	Austria	2002–2014	RCC	430	65.5	59.80%	40	145	NR	NR	yes	7

NR: not reported.

### Quality assessment

While there was a small variation in the methodological quality of included studies, all of the included studies were judged relatively high quality according to the NOS assessment tool, with scores from 6 to 8.

### PLR and survival in patients with urological cancers

**Figure 2:**
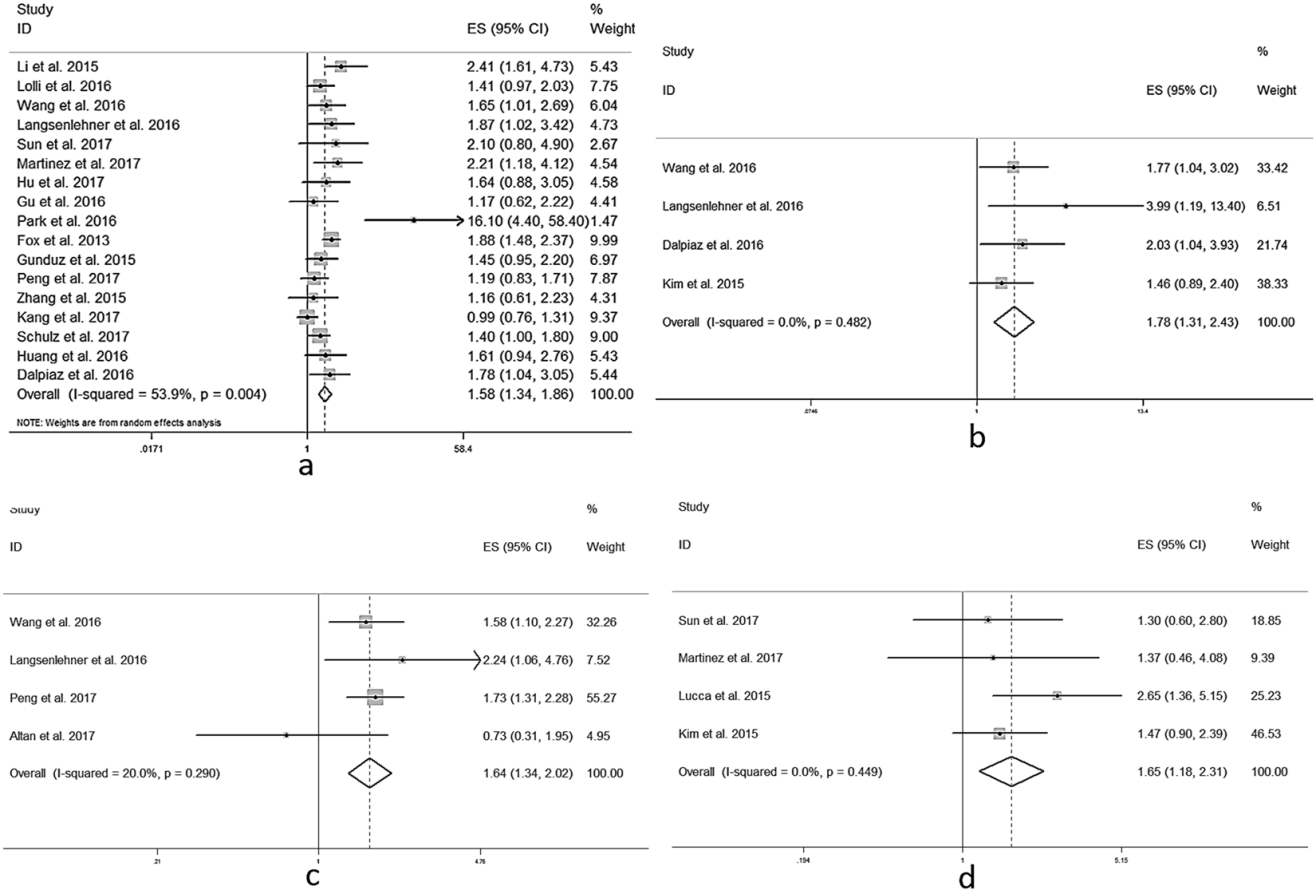
Forest plot of pooled HR of PLR in predicting survival outcomes in urological cancers. (**a**) PLR and OS. (**b**) PLR and CSS. (**c**) PLR and PFS. (**d**) PLR and DFS.

As displayed in Fig. 2a, the forest plot showed high PLR was significantly associated with poor OS in patients with urological cancers. The pooled HR was 1.58 (95% confidence interval [CI]: 1.34–2.86, *P* < 0.001) from 17 studies. In addition, increased PLR was also significantly correlated with poor CSS (pooled HR = 1.78, 95%CI: 1.31–2.43, Fig. 2b). Elevated PLR was significantly associated with poor PFS (pooled HR = 1.64, 95%CI: 1.34–2.02, Fig. 2c). Furthermore, high PLR was significantly associated with poor DFS (pooled HR = 1.65, 95%CI: 1.18–2.31, Fig. 2d) in patients with urological cancers. The above pooled results were not influenced whether using univariate or multivariate HRs separately (Supplementary Table 1, Supplementary Table 2, Supplementary Figure 1).

**Figure 3:**
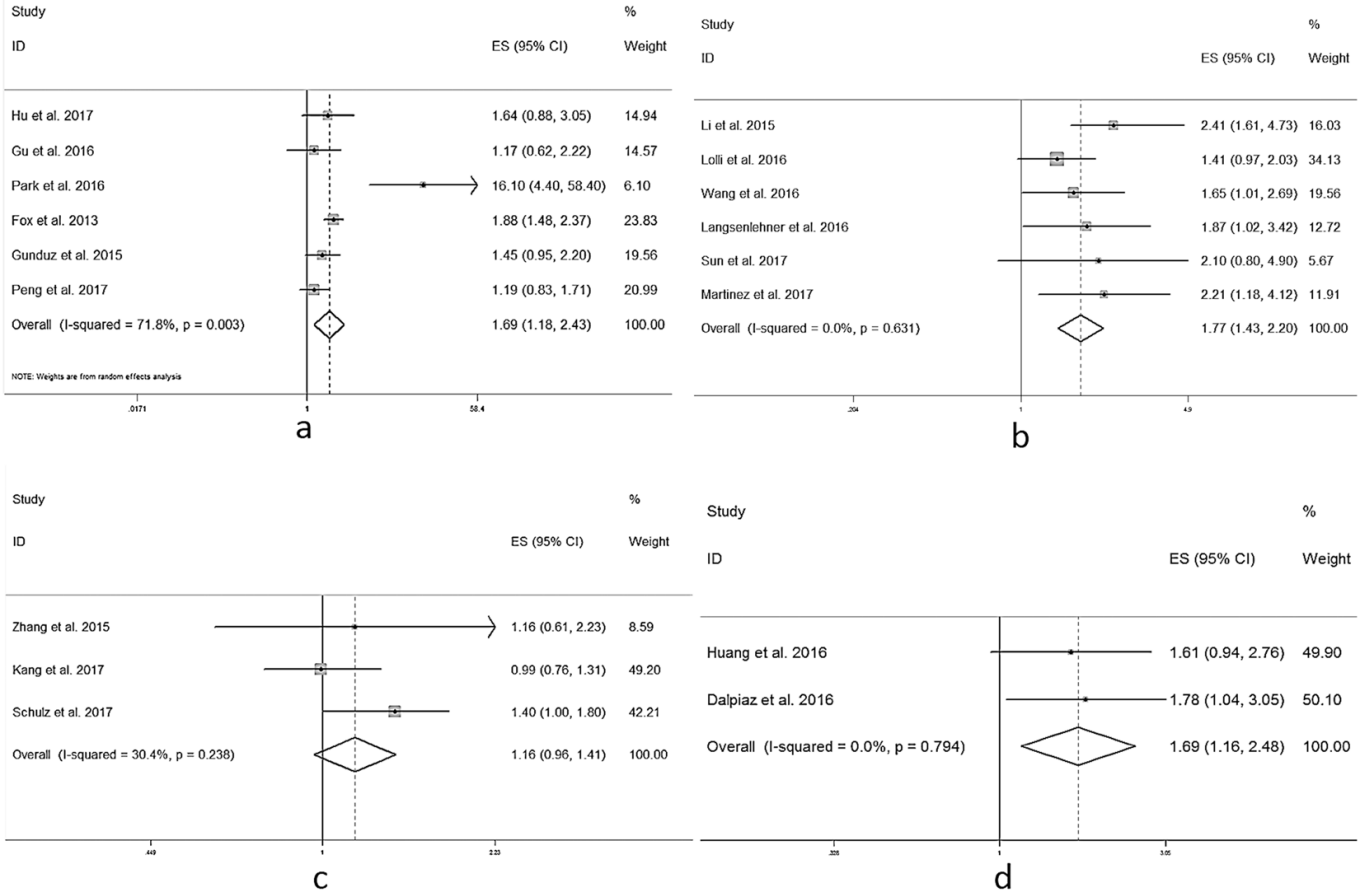
Forest plots of pooled HR of PLR in predicting OS in different types of urological cancers. (**a**) PLR in RCC. (**b**) PLR in PCa. (**c**) PLR in BCa. (**d**) PLR in UTUC.

Then we performed further analyses based on the type of urological cancers. 6 cohorts investigated PLR and OS in patients with RCC (Fig. 3a). The pooled HR was 1.69 (95%CI: 1.18–2.43, *P* = 0.003). While six studies provided HRs of PLR and OS in patients with PCa (Fig. 3b). Their pooled HR was 1.77 (95%CI: 1.43–2.20, *P* < 0.001) and no heterogeneity existed (*I* ^*2*^ = 0%, *P* = 0.631). However, other 3 cohorts reported PLR and OS in patients with BCa (Fig. 3c), whose pooled HR was 1.16 (95%CI: 0.96–1.41, *P* = 0.124), with low heterogeneity existed (*I* ^*2*^ = 30.4%, *P* = 0.238). Only 2 cohorts explored PLR and OS in patients with UTUC (Fig. 3d), whose pooled HR was 1.69 (95%CI: 1.16–2.48, *P* = 0.007). No heterogeneity was found in UTUC group (*I* ^*2*^ = 0%, *P* = 0.794).

### Subgroup analysis and Meta-regression

**Figure 4:**
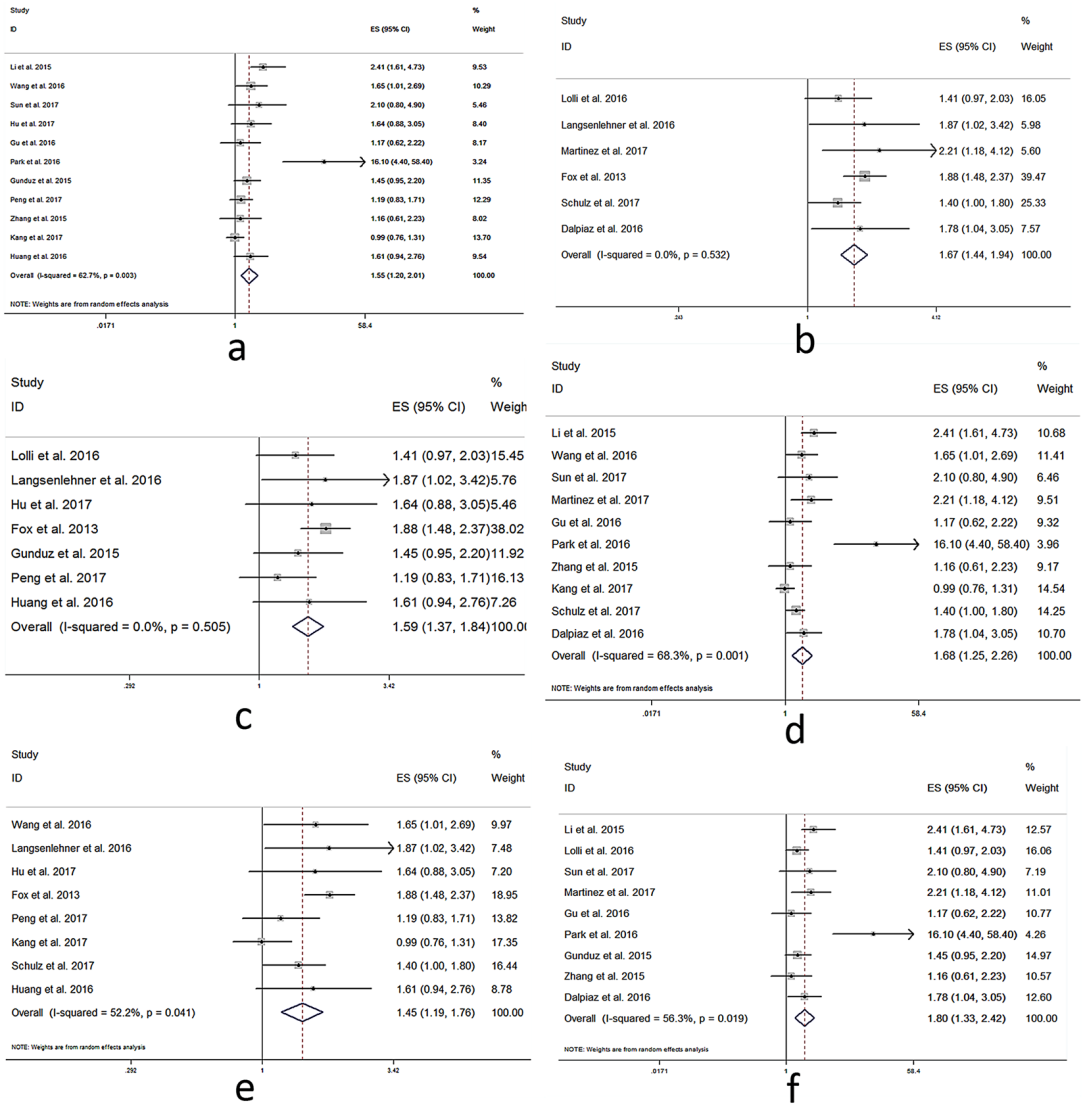
Forest plots of pooled HR of PLR in predicting OS in different subgroups. (**a**) Asains. (**b**) Non-Asians. (**c**) PLR value > 150. (**d**) PLR value ≤ 150. (**e**) Sample size > 254. (**f**) Sample size ≤ 254.

There was slight heterogeneity (Fig. 2a, *I* ^*2*^ = 53.9%), so we conducted subgroup analysis to seek more information (Table 2). In epidemiological studies, ethnicity difference was usually recognized as a critical source of heterogeneity. Notably, the *I* ^2^ values of Asian and non-Asian were 62.7% and 0.0% respectively. From another aspect, there were also no heterogeneity when cut-off value of PLR > 150 (*I* ^2^ = 0.0%). When we conducted subgroup on sample size, heterogeneity existed when sample size > 254 (*I* ^*2*^ = 52.2%) or ≤254 (*I* ^*2*^ = 56.3%). The pooled HR values and their 95%CI in each subgroup analysis were demonstrated in Table 2 with corresponding forest plots shown in Fig. 4. To sum up, the pooled HRs indicated that high PLR was significantly associated with poor OS in each subgroup.

**Table 2:** Summary of the subgroup analysis results of PLR on OS.

Variable	Number of studies	Number of patients	Model	Outcome (OS)		Heterogeneity	
				HR (95%CI)	*P* value	I-square (%)	*P* value
Ethnicity							
Asian	11	4830	R	1.552 (1.196–2.014)	0.001	62.7	0.003
Non-Asian	6	1912	F	1.674 (1.444–1.941)	<0.001	0	0.532
PLR cut-off							
>150	7	3391	F	1.588 (1.374–1.836)	<0.001	0	0.505
≤150	10	3351	R	1.684 (1.254–2.262)	0.001	68.3	0.001
Sample size							
>254	8	5567	R	1.450 (1.192–1.764)	<0.001	52.2	0.041
≤254	9	1175	R	1.796 (1.333–2.420)	<0.001	56.3	0.019

F: fixed-effects model; R: random-effects model.

To seek possible sources of heterogeneity, we also conducted meta-regression. On account of insufficient data on other parameters such as tumor grade or stage, we choose cancer types, PLR cut-off, sample size, follow up time as covariates to estimate between-study variance. The result of this model (*P* = 0.86) showed no potential association between these covariates (cancer types: *P* = 0.367, PLR cut-off: *P* = 0.626, sample size: *P* = 0.254, follow up time: *P* = 0.563) and pooled HR.

### Sensitivity analysis

**Figure 5:**
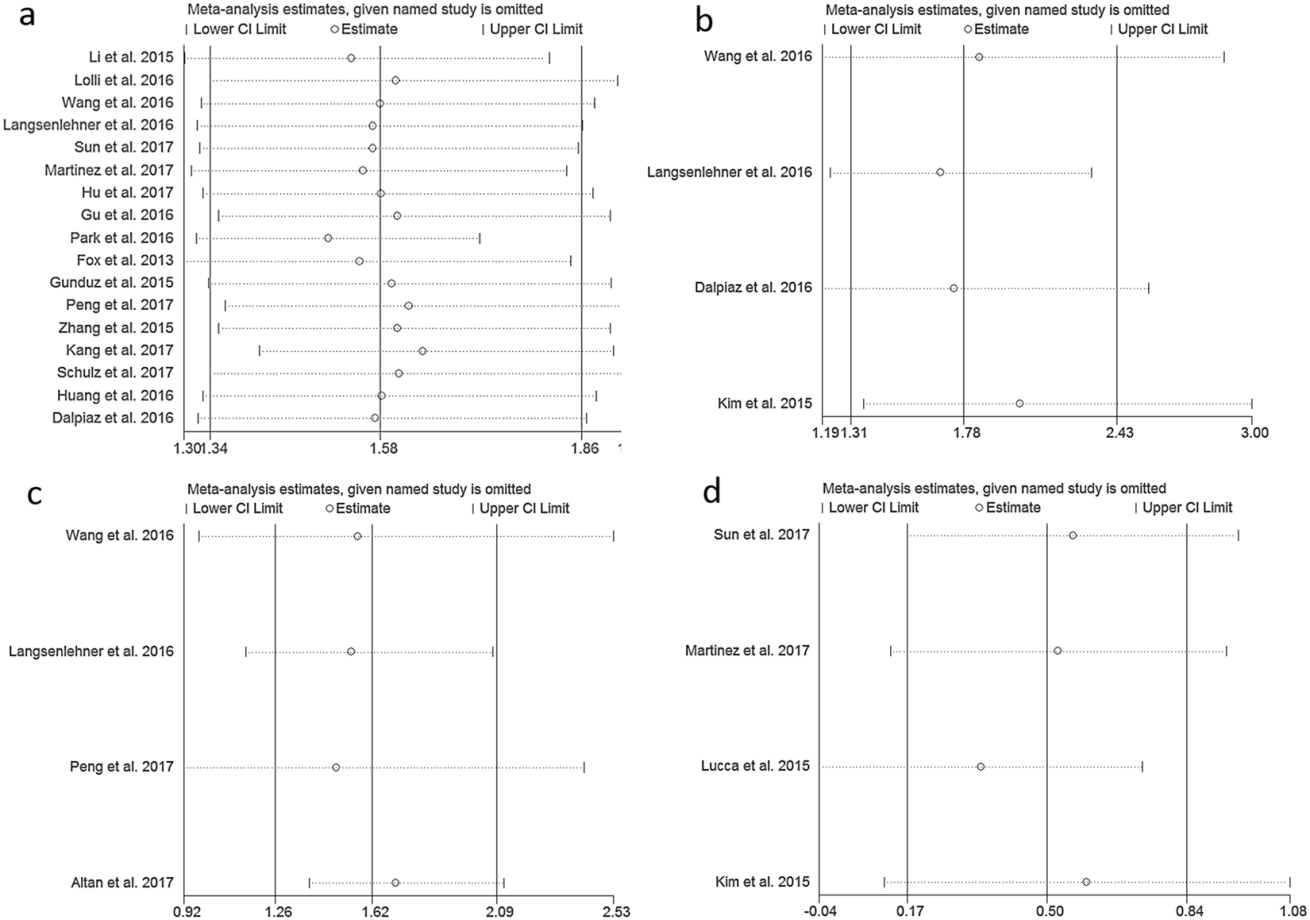
Sensitivity analysis of included studies. (**a**) PLR and OS. (**b**) PLR and CSS. (**c**) PLR and PFS. (**d**) PLR and DFS.

In order to gauge the stability of the results, we conducted sensitivity analysis by removing one study in sequence to see if a single study could have significant impact on the pooled HRs for survival. The results were not significantly altered by removing anyone of the included studies (Fig. 5a: OS, Fig. 5b: CSS, Fig. 5c: PFS, Fig. 5d: DFS).

### Publication bias

**Figure 6:**
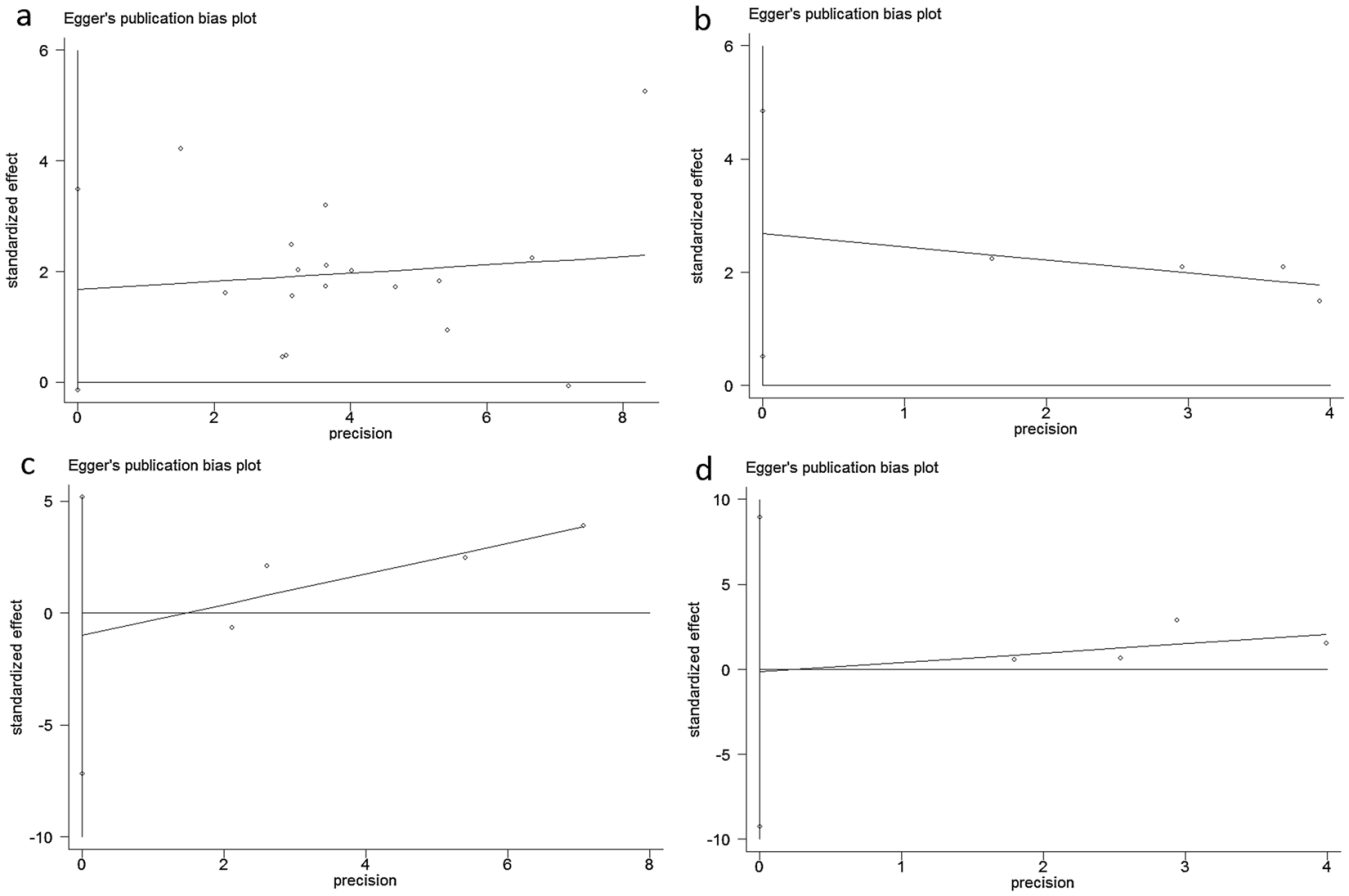
Plots of Egger’s test. (**a**) PLR and OS. (**b**) PLR and CSS. (**c**) PLR and PFS. (**d**) PLR and DFS.

The funnl plots of Egger’s test were displayed in Fig. 6. Both Begg’s test and Egger’s revealed no significant publication bias in this meta-analysis about PLR and OS (Fig. 6a: Begg’s test: Z value = 1.73, <1.96; *P* value = 0.091, >0.05; Egger’s test: *P* value = 0.068, >0.05), CSS (Fig. 6b: Begg’s *P* value = 0.089; Egger’s *P* value = 0.033), PFS (Fig. 6c: Begg’s *P* value = 0.734; Egger’s *P* value = 0.557) and DFS (Fig. 6d: Begg’s *P* value = 0.999; Egger’s *P* value = 0.952).

## Discussions

Currently, no standard quantitative biomarkers are perfect enough to assess the clinical outcomes in patients with urological cancers. According to the Food and Drug Administration, a clinical validating biomarker should be measured reproducible and consistently^37^. Blood parameters, like NLR and PLR, are convenient and easy to be acquired during routinely clinical practice.

In our current meta-analysis, we utilized the existing evidence from 14 included studies to obtain the pooled results that an elevated pretreatment PLR indicated unfavorable worse OS (HR = 1.58, 95%CI: 1.34–1.86) among patients with urological cancers. High pretreatment PLR was also correlated with poor CSS (HR = 1.78, 95%CI: 1.31–1.43), PFS (HR = 1.64, 95%CI: 1.34–2.02) and DFS (HR = 1.65, 95%CI: 1.18–2.31), with no heterogeneity.

Notably, this correlation of high PLR and poor OS was also applicable to RCC, PCa and UTUC, but not in BCa (HR = 1.16, 95%CI: 0.96–1.41). Only 3 studies available for meta-analysis on PLR and OS in patients with BCa, so it should be interpreted cautiously.

Subgroup analysis divided by ethnicity, sample sizes and PLR cut-off value did not significantly change the main results. Taken all the above results into consideration, we believed that PLR could serve as an reliable marker in urological prognostication. PLR, an parameter which is reproducible, convenient and low cost, can be available from blood routine test in daily clinical practice. Additionally, PLR changes followed by tumor changes during anticancer process are also crucial to know for its application as an indicator of treatment efficacy.

Our findings about PLR are in accordance with previous reported other cancers such as breast cancer, lung cancer and colorectal cancer^38–40^. Systemic inflammatory plays a crucial role in tumor progression at almost every single step including initiation, progression, and metastasis^41^. But the underlying mechanism that PLR influence the survival of patients with urological cancers remains largely unknown. Several hypotheses have been put forward to explain this phenomenon. Platelet-derived cytokines, such as platelet derived growth factor (PDGF), vascular endothelial growth factor (VEGF) and basic fibroblast growth factor (bFGF) have been found to regulate and promote tumor angiogenesis, then further accelerate tumor aggressiveness^42,43^. Platelet can also release

microparticles that help to guard tumor cells from the elimination of natural killer^44^. On the contrary, the reduction of lymphocytes was correlated with poor prognosis, as indicated in a previous study^45^. Lymphocytes, like T-lymphocytes who are able to secret interleukin-4 and -5 in tumor microenvironment, have an anti-tumor activity by inducing cytotoxic cell death and inhibiting tumor proliferation^46,47^. In conclusion, the platelet to lymphocyte ratio could be regarded as a positive correlative marker with worse cancer prognosis theoretically.

Although this is the first meta-analysis concerning PLR and urological cancer prognostication, several limitations are still needed to be addressed. Firstly, though we didn’t restricted language, all the 20 studies included were published in English. We found no relevant studies published in Chinese qualified to the inclusion criteria, but we also failed to identify articles in other languages, which might lead to little language bias. Secondly, although sensitivity analysis supported the stability of our results and no publication bias found, the number of studies was relatively small in different cancer types (6 for RCC, 6 for PCa, 3 for BCa, 2 for UTUC) and in some subgroups (6 for non-Asians). Thus we wish to emphasize that the results should be cautiously interpreted. Thirdly, heterogeneity among studies was found, which was probably because of the relatively small sample sizes and the retrospective property of study design. Fourthly, we lack the PLR data of patients with comprehensive different urological cancer stages (localized, advanced or metastasis) at present. Large scale statistics about PLR response during different treatment strategies such as radiotherapy, chemotherapy and immunotherapy are also insufficient. So further large prospective clinical trials are urgently required to verify the prognostic value of PLR in patients with urological cancers in the future.

In summary, this meta-analysis suggested that elevated PLR was negatively related to survival of patients with urological cancers, except in BCa. However, further high quality studies with large sample size should be conducted to validate this paper’s results.

## Methods

### Search strategy

This meta-analysis was conducted under the guidelines of the Preferred Reporting Items for Systematic Reviews and Meta-Analyses (PRISMA)^48^. A comprehensive literature search for relevant studies in the PubMed, Embase and Web of Science was performed through July 24th, 2017. The searching strategy consisted of medical subheadings and key words. The main terms were as follows: (‘urological neoplasms[MeSH]’ OR ‘prostate cancer’ OR ‘bladder cancer’ OR ‘renal cell carcinoma’ OR ‘renal cancer’ or ‘urinary tract cancer’ OR ‘upper urinary tract urothelial carcinoma’) AND (‘platelet to lymphocyte ratio’ OR ‘PLR’) AND (‘prognosis[MeSH]’ OR ‘survival’ OR ‘outcome’). The language of studies, population and sample size were not restricted. We also manually searched the reference lists for additional relevant publications.

## Study Selection

### Inclusion and exclusion criteria

Studies meeting the following criteria were considered eligible:1. clinical cohort evaluated the prognostic accuracy of PLR in urological cancers; 2. studies compared PLR with other prognosis models and reported survival outcomes like OS and PFS; 3. reported original hazard ratio (HR) with 95% confidence interval (95%CI) or HR could be extracted from sufficient information; 4. articles with the most complete information and the largest cohort if there were multiple studies by the same author or institute.

The exclusion criteria were: 1. repeated publications; 2. studies reporting on less than 20 patients; 3. experimental laboratory articles, animal studies, letters or review articles.

### Assessment of study quality

Two investigators(DY.L. and XY.H.) independently reviewed all relevant articles and judged the methodology quality of potential studies using Newcastle–Ottawa Quality Assessment Scale (NOS) assessment tool, including selection, comparability and outcome^49^. A study was considered high quality if the NOS score ≥7. When disagreements occurred, the two reviewers reached consensus by involving a third author(HX.D.).

### Data extraction

We extracted the following variables from each study: first author’s name; publication year; study design; country or region of the study; type of urological cancer; cut-off value of PLR; sample size; age, sex, out-come assessment and risk estimates, follow up time and HRs with 95%CI. If the HRs of both univariate and multivariate analysis for the same comparison were available, we only used the latter for analysis. We also used univariate or multivariate HRs separately to test methodological sensitivity. If the HR and 95%CI were not displayed directly, they were estimated from Kaplan–Meier curves^50^. If necessary, the corresponding author was contacted for further information.

### Statistical analysis

HRs with 95%CI were pooled using a meta-analysis to access the strength of PLR to survival endpoints such as OS, CSS, PFS and DFS. All the PLR values from 20 included studies were categorical variables in survival analysis. The Cochrane Q test was used to determine the heterogeneity among studies. A *P* value < 0.10 indicated heterogeneity. Inconsistency (*I* ^*2*^) was also calculated to evaluate heterogeneity. An*I* ^2^ value > 50% was considered significant heterogeneity. The fixed-effect model (inverse variance method) was used to calculate pooled results when no heterogeneity existed among included studies, otherwise, a random-effect model (DerSimonian and Laird method) was used with the weights inversely proportional to the variance of log hazard ratio of each trial^51,52^. To find reasons of heterogeneity among studies, we conducted subgroup analysis in ethnic difference, different cut-off value and sample size respectively. When the log-rank statistical value was maximum on receiver operating characteristic curve, the cut-off value of PLR was decided. The median value of sample size was chosen to divide subgroup. Meta-regression was also performed by using cancer type, PLR cut-off, sample size and follow up time as covariates. To test the reliability of the main outcomes in our analysis, sensitivity analysis was performed by removing one single study in turn. Egger’s and Begg’s tests with funnel plots were used to test publication bias. *P* value > 0.05 indicated no potential publication bias. Kaplan–Meier curves were read by Engauge Digitizer version 9.8 (http://markummitchell.github.io/engauge-digitizer/). We used Stata 12.0 software (Stata Corporation, College Station, TX, USA) to conduct all the statistical analyses. A two-sided *P* value less than 0.05 was considered statistically significant.

### Data availability

In our current meta-analysis, all original data analyzed were derived from published articles. All data generated during this study are included in the present article.

## Electronic supplementary material

Supplementary Information
