# Supplementary material for: The Prognostic Value of Platelet-to-Lymphocyte Ratio in Urological Cancers: A Meta-Analysis
Source: Sci Rep. 2017 Nov 13;7:15387. doi: 10.1038/s41598-017-15673-2 (PMC5684392; doi:10.1038/s41598-017-15673-2)
Supplement: Supplementary file 1 — Supplementary Information [file 41598_2017_15673_MOESM1_ESM.pdf]

The Prognostic Value of Platelet-to-Lymphocyte Ratio in Urological Cancers: A Meta-Analysis

Dong-Yang Li, Xuan-Yu Hao, Tian-Ming Ma, Hui-Xu Da, Yong-Sheng Song

Supplementary Table 1. HR values and 95%CI of different survival outcomes from included studies.

Study ID	HR of OS (UNI)	95% CI	HR of OS (MULTI)	95% CI	HR of CSS (UNI)	95% CI	HR of CSS (MULTI)	95% CI	HR of PFS (UNI)	95% CI	HR of PFS (MULTI)	95% CI	HR of DFS (UNI)	95% CI	HR of DFS (MULTI)	95% CI
Li et al. 2015	2.67	1.73-4.92	2.41	1.61-4.73	NR	NR	NR	NR	NR	NR	NR	NR	NR	NR	NR	NR
Lolli et al. 2016	1.68	1.20–2.35	1.41	0.97–2.03	NR	NR	NR	NR	NR	NR	NR	NR	NR	NR	NR	NR
Wang et al. 2016	1.781	1.096–2.894	1.65	1.013–2.687	1.948	1.145–3.312	1.768	1.036–3.015	1.48	1.040–2.107	1.581	1.100–2.272	NR	NR	NR	NR
Langsenlehner et al. 2016	1.76	1.07-2.91	1.87	1.02-3.42	3.71	1.41–9.74	3.99	1.19–13.4	2.4	1.31–4.39	2.24	1.06–4.76	NR	NR	NR	NR
Sun et al. 2017	2.1	0.8-4.9	2.1	0.8-4.9	NR	NR	NR	NR	NR	NR	NR	NR	1.9	1.0-3.5	1.3	0.6-2.8
Martinez et al. 2017	2.21	1.18-4.12	2.21	1.18-4.12	NR	NR	NR	NR	NR	NR	NR	NR	NR	NR	1.37	0.46-4.08
Hu et al. 2017	4.89	3.10–7.72	1.64	0.88–3.05	NR	NR	NR	NR	NR	NR	NR	NR	NR	NR	NR	NR
Gu et al. 2016	2.386	1.482–3.840	1.172	0.618–2.220	NR	NR	NR	NR	NR	NR	NR	NR	NR	NR	NR	NR
Park et al. 2016	13.7	7.8–19.6	16.1	4.4–58.4	NR	NR	NR	NR	NR	NR	NR	NR	NR	NR	NR	NR
Fox et al. 2013	1.88	1.48-2.37	1.88	1.48-2.37	NR	NR	NR	NR	NR	NR	NR	NR	NR	NR	NR	NR
Gunduz et al. 2015	NR	NR	1.445	0.949-2.200	NR	NR	NR	NR	NR	NR	NR	NR	NR	NR	NR	NR
Peng et al. 2017	1.19	0.83-1.71	1.19	0.83-1.71	NR	NR	NR	NR	NR	NR	NR	NR	1.73	1.31-2.28	NR	NR
Zhang et al. 2015	1.161	0.605–2.226	1.161	0.605–2.226	NR	NR	NR	NR	NR	NR	NR	NR	NR	NR	NR	NR
Kang et al. 2017	NR	NR	0.99	0.76-1.31	NR	NR	NR	NR	NR	NR	NR	NR	NR	NR	NR	NR

Schulz et al. 2017	NR	NR	1.4	1.0-1.8	NR	NR	NR	NR	NR	NR	NR	NR	NR	NR	NR	NR
Huang et al. 2016	NR	NR	1.61	0.94-2.76	NR	NR	NR	NR	NR	NR	NR	NR	NR	NR	NR	NR
Dalpiaz et al. 2016	2.699	1.639–4.443	1.782	1.041–3.050	3.035	1.627–5.664	2.026	1.045–3.930	NR	NR	NR	NR	NR	NR	NR	NR
Kim et al. 2015	NR	NR	NR	NR	1.46	0.887–2.405	NR	NR	NR	NR	NR	NR	1.466	0.898–2.394	NR	NR
Altan et al. 2017	NR	NR	NR	NR	NR	NR	NR	NR	NR	NR	0.733	0.306-1.951	NR	NR	NR	NR
Lucca et al. 2015	NR	NR	NR	NR	NR	NR	NR	NR	NR	NR	NR	NR	NR	NR	2.65	1.36–5.15

NR: not reported; UNI: univariate analysis; MULTI: multivariate analysis; OS: overall survival; CSS: cancer specific survival; PFS: progression free survival; DFS: disease free survival .

Supplementary Table 2. Summary of the meta-analysis results of PLR on different survival outcomes using univariate or multivariate HRs separately.

			Meta-analysis results		Heterogeneity	
Variable	Number of studies	Model	HR (95%CI)	*P* value	I-square (%)	*P* value
Outcome (OS)						
Univariate	13	R	2.375 (1.676 - 3.367)	<0.001	87.2	<0.001
multivariate	12	R	1.585 (1.279 - 1.963)	<0.001	58.6	0.005
Outcome (CSS)						
Univariate	4	F	1.881 (1.390 - 2.546)	<0.001	0	0.399
multivariate	3	F	2.021 (1.364 - 2.995)	0.001	0	0.483
Outcome (PFS)						
Univariate	3	F	1.704 (1.388- 2.092)	<0.001	0	0.395
multivariate	3	F	1.540 (1.132- 2.095)	0.006	41.9	0.179
Outcome (DFS)						
Univariate	2	F	1.618 (1.100 - 2.380)	0.015	0	0.523
multivariate	3	F	1.836 (1.162 - 2.901)	0.009	9.7	0.330

F: fixed-effects model; R: random-effects model.

Supplementary Figure 1. Forest plots of pooled HR of PLR in predicting survival in different urological cancers. a. Univariate PLR and OS. b. Univariate PLR and CSS. c. Univariate PLR and PFS. d. Univariate PLR and DFS. e. Multivariate PLR and OS. f. Multivariate PLR and CSS. g. Multivariate PLR and PFS. h. Multivariate PLR and DFS.

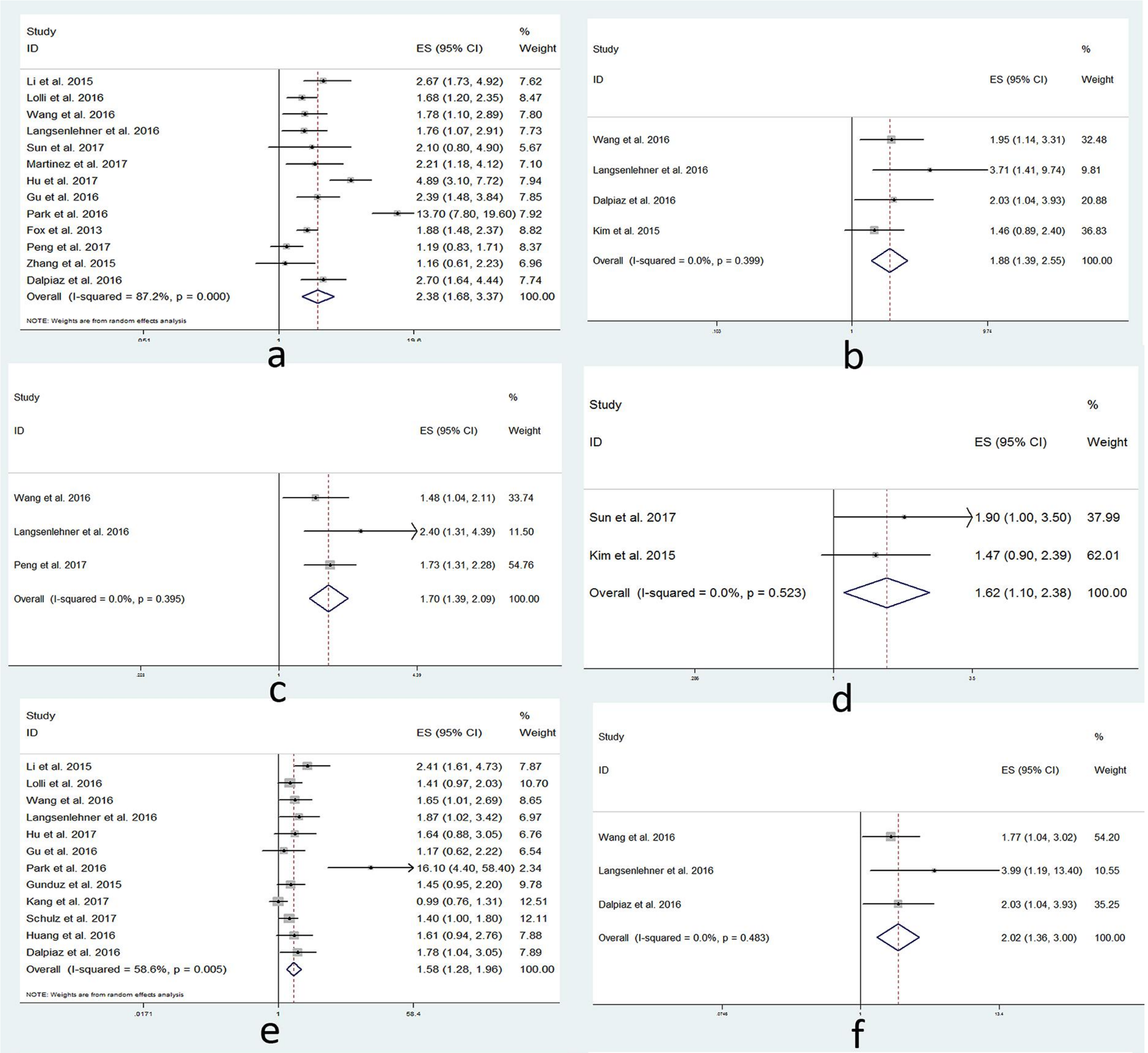

Study
ID
ES (95% CI)
%
Weight
Li et al. 2015 2.67 (1.73, 4.92) 7.62
Lolli et al. 2016 1.68 (1.20, 2.35) 8.47
Wang et al. 2016 1.78 (1.10, 2.89) 7.80
Langsenlehner et al. 2016 1.76 (1.07, 2.91) 7.73
Sun et al. 2017 2.10 (0.80, 4.90) 5.67
Martinez et al. 2017 2.21 (1.18, 4.12) 7.10
Hu et al. 2017 4.89 (3.10, 7.72) 7.94
Gu et al. 2016 2.39 (1.48, 3.84) 7.85
Park et al. 2016 13.70 (7.80, 19.60) 7.92
Fox et al. 2013 1.88 (1.48, 2.37) 8.82
Peng et al. 2017 1.19 (0.83, 1.71) 8.37
Zhang et al. 2015 1.16 (0.61, 2.23) 6.96
Dalpiaz et al. 2016 2.70 (1.64, 4.44) 7.74
Overall (I-squared = 87.2%, p = 0.000) 2.38 (1.68, 3.37) 100.00
NOTE: Weights are from random effects analysis
1
a
Study
ID
ES (95% CI)
%
Weight
Wang et al. 2016 1.95 (1.14, 3.31) 32.48
Langsenlehner et al. 2016 3.71 (1.41, 9.74) 9.81
Dalpiaz et al. 2016 2.03 (1.04, 3.93) 20.88
Kim et al. 2015 1.46 (0.89, 2.40) 36.83
Overall (I-squared = 0.0%, p = 0.399) 1.88 (1.39, 2.55) 100.00
1
b
Study
ID
ES (95% CI)
%
Weight
Wang et al. 2016 1.48 (1.04, 2.11) 33.74
Langsenlehner et al. 2016 2.40 (1.31, 4.39) 11.50
Peng et al. 2017 1.73 (1.31, 2.28) 54.76
Overall (I-squared = 0.0%, p = 0.395) 1.70 (1.39, 2.09) 100.00
1
c
Study
ID
ES (95% CI)
%
Weight
Sun et al. 2017 1.90 (1.00, 3.50) 37.99
Kim et al. 2015 1.47 (0.90, 2.39) 62.01
Overall (I-squared = 0.0%, p = 0.523) 1.62 (1.10, 2.38) 100.00
1
3.5
d
Study
ID
ES (95% CI)
%
Weight
Li et al. 2015 2.41 (1.61, 4.73) 7.87
Lolli et al. 2016 1.41 (0.97, 2.03) 10.70
Wang et al. 2016 1.65 (1.01, 2.69) 8.65
Langsenlehner et al. 2016 1.87 (1.02, 3.42) 6.97
Hu et al. 2017 1.64 (0.88, 3.05) 6.76
Gu et al. 2016 1.17 (0.62, 2.22) 6.54
Park et al. 2016 16.10 (4.40, 58.40) 2.34
Gunduz et al. 2015 1.45 (0.95, 2.20) 9.78
Kang et al. 2017 0.99 (0.76, 1.31) 12.51
Schulz et al. 2017 1.40 (1.00, 1.80) 12.11
Huang et al. 2016 1.61 (0.94, 2.76) 7.88
Dalpiaz et al. 2016 1.78 (1.04, 3.05) 7.89
Overall (I-squared = 58.6%, p = 0.005) 1.58 (1.28, 1.96) 100.00
NOTE: Weights are from random effects analysis
.0171
1
58.4
e
Study
ID
ES (95% CI)
%
Weight
Wang et al. 2016 1.77 (1.04, 3.02) 54.20
Langsenlehner et al. 2016 3.99 (1.19, 13.40) 10.55
Dalpiaz et al. 2016 2.03 (1.04, 3.93) 35.25
Overall (I-squared = 0.0%, p = 0.483) 2.02 (1.36, 3.00) 100.00
1
f
